# Effects of a selected exercise programon executive function of children with attention deficit hyperactivity disorder

**Published:** 2016

**Authors:** M Memarmoghaddam, HT Torbati, M Sohrabi, A Mashhadi, A Kashi

**Affiliations:** * Department of Motor Behavior, Faculty of Physical Education and Sport Sciences, Ferdowsi University Of Mashhad, Mashhad, Iran; ** Department of Psychology, Faculty of Education and Psychology, Ferdowsi University of Mashhad, Mashhad, Iran; *** Sport Sciences Research Institute of Iran, Tehran, Iran

**Keywords:** Attention Deficit Hyperactivity Disorder (ADHD), Selective exercise program, Executive Function

## Abstract

**Introduction.** The aim of this study was to examine the effectiveness of a Selected exercise program on the executive function of children with ADHD.

**Method.** The participants were 40 male students, aged 7-11 years. The participants were randomly assigned into two groups (experimental and control). The experimental group participated in an exercise program for 24 sessions, 90 minutes per session. The control group did not receive any intervention. Before and after the exercise period, all the participants were assessed with Stroop and Go-No-Go tests, and the resulting data were analyzed by using MANCOVA.

**Result.** The results showed that the cognitive inhibition of the children in the experimental group was significantly different compared with the control group (p < 0.05). Additionally, there was a significant difference between the experimental and control groups in the behavioral inhibition (p < 0.05).

**Conclusion.** An organized physical activity helps to improve the executive function in children with ADHD.

## Introduction

Attention deficit hyperactivity disorder (ADHD) is one of the most common neurodevelopmental disorders in childhood. There are three sub-types of ADHD: predominantly inattentive subtype (ADHD-I), ADHD predominantly hyperactive/impulsive subtype (ADHD-H) andADHD combined subtype (ADHD-C) [**1**]. This disorder affects approximately 3 to 5% of school-age children, and boys are 3 to 7 times more likely to be affected by this disorder than girls are [**2**]. In 60 to 80% of these patients, this disorder continues to adolescence and adulthood, which can result in educational / occupational challenges, difficult relationships, anxiety, depression, aggression, lawlessness, and substance abuse [**3**]. Studies indicate that children with ADHD often have defects in cognitive performance, especially in the executive function (EF). EF includes all complex cognitive processes that are needed to perform new or difficult tasks. EFis dependent on brain function, especially of the prefrontal cortex (PFC) [**4**]. One of the most important components of EFis inhibition that is both cognitive and behavioral. The cognitive inhibition prevents the entrance of irrelevant information about a task to the working memory [**5**]. However, in behavioral inhibition, a person’s ability to stop or delay an activity is considered. In other words, the aim of this process is to control physical behaviors, particularly the prevention of unwanted behaviors and reactions [**6**]. ADHD treatment methods are divided into two groups, drug and non-drug treatments. However, due to the side effects of the stimulus drugs, such as loss of appetite, insomnia, or personality changes, it is necessary to find an alternative treatment [**3**]. A growing research literature suggests that physical activity and exercise can play an important role in managing the symptoms of ADHD [**3**,**7**,**8**]. Studies report that physical activity and exercise can result in beneficial changes in EF by stimulating the neurobiological processes [**9**]. Moreover, a recent review of several large-scale experimental studies suggests that physical activity training exerts specific effects on cognitive functions of children from the general population [**10**]. Those results are especially interesting for children with ADHD in the ADHD theoretical model of Barkley (1997),which suggests that inhibition is the principal deficit of this disorder. This inhibition deficit impedes four executive neuropsychological functions: working memory, self-regulation of affect, internalization of speech, and reconstitution leading to problems of behavioral self-regulation (inattention, hyperactivity-impulsivity). Therefore, if physical activity can improve inhibition and the executive functions, one could expect an improvement of self-regulation [**11**].However, few studies have been conducted on the impact of physical activity on the inhibitory executive functions, especially in children with ADHD, therefore, inconsistent results emerge. Smith et al. (2013) examined the effect of an 8-week physical activity on EF. The results showed significant changes in the attention and response inhibition [**12**]. Verret et al. (2012) examined the effects of a physical activity program on the cognitive performance of children with ADHD, but the results of the response inhibition test were not significant [**13**]. In contrast,another study showed that a five minutes jumping on a trampoline (using a Go-No-Go test) has a positive effect on the inhibition in children with ADHD [**14**]. Further, Pan et al. (2015) reported that twelve weeks of tennis exercise had an effect on the scores of the color-word condition of the Stroop test [**15**]. Other researchers have shown the effect of acute exercise, only on some components of cognitive inhibition [**16**-**18**]. Also, researchers have shown, the effect of 30 minutes running on a treadmill [**19**], and an aquatic exercise program for 8 weeks on certain components of Go-No-Go test [**20**].

Generally, although the physical activity is an acceptable treatment for children with ADHD, there is a limited evidence on EFchildren, especially inhibition in children with ADHD. However, some of these results are inconsistent, with a small sample size, heterogeneity characterization of samples, or without a control group, that does not allow a definitive consensus. Researchers have suggested that due to a lack of consideration of important factors (e.g. medications, comorbid conditions, and the economic situation), better and higher-quality evidence is needed to firmly show the extent of the positive effects of exercise on children with ADHD [**3**,**7**,**21**,**22**]. The relationship between exercise and cognition in children depends on several modulators, such as age, type of activity and exercise, intensity, duration and frequency of exercise, and types of cognition. Thus, it has been suggested that future research should focus on the relationship between exercise and cognitive parameters in patients with this disorder in order to create a foundation for exercise as a therapeutic method in this disorder [**21**,**22**]. Designing new interventions in childhood and growth age to increase the neurological and cognitive development, especially in the areas of the brain that are Intermediate EF, may be more effective and sustainable than the current interventions (such as medicine) and cause stable improvements in the severity of ADHD symptoms during life [**3**]. Researchers believe that creating exercise programs, which can have an impact on children with attention deficits and keep them involved, provides a future research challenge [**3**,**22**].

On the other hand, in previous studies, the various types of inhibition (cognitive and behavioral inhibition) were not examined separately, so that it is difficult to indicate the role of physical activity on each type of inhibition. The researchers suggest that further research is encouraged to emphasize inhibition, which is the main symptom of ADHD patients. Regarding inhibition, other research has further distinguished“behavioral inhibition” and “resistance to interference” from “cognitive inhibition”, an active suppression of cognitive content that is currently executed in the working memory[**23**]. 

So, in this study, an attempt was made to control the intensity, duration, frequency, and type of exercise training to conduct an exercise program that will have the greatest efficacy on the EF in children with ADHD,and to seek the answer to the question whether a selective exercise program can have an impact on cognitive function, particularly cognitive inhibition and behavior.

## Method

Participants

The research population included boys, primary school students (aged from 7 to 11 years), from the sixth educational district. This study was interventional, thus a voluntary sampling method was used. The children suspected of having ADHD were identified with the help of teachers and health educators. After contacting their parents and completing the SNAP-IV rating scale and the Child Behavior Checklist (CBCL) by the parents, students with a confirmed diagnosis of ADHD were introduced to a psychiatrist specialized in children and adolescents. The exclusion criteria of the study sample included an IQ score below 70, children with autism, and other mental disorders and health problems. None of the studied participants used any drug, because one of the goals in this study was to investigate the pure effect of exercise on children in the absence of drug use and the direct benefits of exercise as a treatment method. Finally, 40 of these students were enrolled in this study. Then, the students were randomly divided into two groups (control and exercise programs).At the end of the study, one member from the experimental group (due to irregular participation) and three members from the control group (due to a lack of participation) were excluded from the post-test. The university’s Institutional Review Board approved this study. A consent was obtained from the children and a written informed consent was obtained from their guardians. **Table 1** shows the participants’ demographic characteristics.

**Table 1:** Participants’ detail demographic characteristics

Variables	Group	
	Physical activity group	Control group
	(n = 19)	(n = 17)
Age (year)ᵃ	8.31±1.29	8.29 ±1.31
Height (in)ᵃ	129±1.13	130±5.72
Weight (lb)ᵃ	28.26±6.41	27.76±3.88
ADHD type (n)		
Hyperactive	6	5
Inattentive	5	3
Combined	8	0
ᵃValues are presented as mean±SD.		

**ADHD diagnosis**

**SNAP IV**

For the diagnosis of ADHD, SNAP-IV rating scale was used, which is a standardized test, based on symptoms of Psychiatric Association of America (DSM IV).

This scale has a questionnaire with 18 questions to be answered by parents and teachers. Nine questions are used to identify the ADHD-I subtype, and nine questions are used for the diagnosis of ADHD-H. Each question was scored from 0-3 [**24**,**25**]. Cronbach’s alpha coefficient of this test is reported to be 97% [**24**].

**Child Behavior Checklist (CBCL)**

CBCL questionnaire for ages 6 to 18 years was used to identify patients and study the comorbid disorders in these children. This questionnaire evaluates the behavioral problems and social competences of these children. Eight scales were calculated: anxiety-depression (13 components), withdrawn-depression (8 components), somatic complaints (11 components), social problems (11 components), thought problems (15 components), attention problems (10 components), rule-breaking behaviors (17 components), and aggressive behaviors (18 components). Their compilation allows a scaled computation of internalized, externalized, and total problems.This questionnaire was completed by parents based on the child’s status in the previous 6 months. The reliability coefficient of this form is well reported (r = 0.85) [**26**]. 

**Clinical Interview**

This study used an interview diagnosis conducted by a psychiatric specialist in children and adolescents.

**Cognitive performance measures**

**Stroop Test**

This test was used to measure selective attention and cognitive flexibility. At present, the Stroop test is predominately used to measure cognitive inhibition. In this study, an electronic version of the test was employed.

When the participant saw each colored circle, he pressed a key with the same color. In the second exercise, the participant (without paying attention to the word itself) chose the appropriate color key for each word that he saw. At the end of the training, participants entered the main stage,which was similar to the second exercise. In this section, the participant observed 48 consistent colored words and 48 inconsistent colored words that appeared randomly in succession on the screen, at this point the participant had tochoose the same color with the color of each word. The validity of the test has been reported to be between 0.80 - 0.91 [**27**].

**Go-No-Go Test**

The aim of the Go-No-Go test was to measure behavior inhibition. The test included two types of consistent and inconsistent stimuli and the participant had to answer to a set of consistent stimuli and avoid answering to inconsistent stimuli. The inability to demonstrate appropriate inhibition or committing errors in this test occurred with a motor response while providing non-target stimuli. In the process of providing stimuli, the number of “Go” stimuli was more than “No Go”. The first few efforts were done for training so that the participants could obtain the necessary knowledge about the test and then answer to 100 major efforts, of which 70 were “Go”. The reliability of this test has been reported to be of 0.87[**28**].

**Exercise-related measures**

**HR**

HRR is an Advisedfor establishing the exercise intensity. HRR was calculated as maximal HR minus resting HR. To assess the resting HR, participants were asked to sit quietly in a chair for a few minutes. Maximal HR was estimated by using the formula 220 - Age. The target HR was calculated by a formula as it follows: Target HR = )HRmax - resting HR) x percentage intensity desired + resting HR [**29**]. In this research, the target heart rate was determined from moderate to Intensive (65% to 80% HRR). This intensity was only controlled in aerobic exercise and competitive exercise programs (about 50 min of the total schedule). To monitor the intensity of the exercise, each participant used a Polar watch (Model FT 4, Polar Electror Oy, Kempele, Finland). An alarm was set on the HR monitor to alert trainers when the HR was either above or below the target rate.

**Selected exercise program**

Selected exercise programincluded a set of physical exercises that aimed to improve EF, specifically in the inhibition of response and behavior. The exercise program was three days a week (24 sessions in 8 weeks) in 90-minute sessions and was conducted at the University Sports Hall. The first 15 minutes of each session were dedicated to warm-up and aerobic exercise.

The next 25 minutes goal-directed exercise, such as using a table tennis racket and balls, targeting the ball to the basket with a variety of sizes and different distances, bowling, hit by rockets on the balance beam, keeping the ball on the racket while walking, jumping into the specified color ring, collecting colored balls, jumping into squares with specific numbers, sit down, get up directly and inverse.The10 minutes Station trainingmeant that there were two types of training in station. The participant was asked to do one exercise only and ignore the other to reach the last station within a short time, 15 min of running on a treadmill in a progressive program,15 minutes ball games, such as football, basketball, etc., for aerobic exercise and to increase motivation and interest to participate in the training sessions, cool-down for 10 minutes.

To ensure the integrity of the Training,Heart rate control, and feedback to the participant, a trained physical education expert was provided for every four individuals. It should be noted that this program was designed under the supervision of physical education professors and before performing this as the main program, it was tested on 10 children with ADHD.

**Procedure**

After the identification and selection of ADHD children and obtaining consent from their parents, these children were asked to visit the University of Motor Behavior Laboratory. Children suffering from ADHD were randomly divided into two groups based on the characteristics of ADHD. Demographic information (e.g., height, weight) were collected. Then, the participants were asked to complete the Stroop test (cognitive inhibition) and the Go-No-Go test (behavioral inhibition). 

The experimental group participated in an exercise program lasting for three sessions of 90 minutes per week for 8 weeks (24 sessions) over a period of two months (July and August 2015) held at the University Sports Hall. After the training sessions, Stroop and Go-No-Go measurements were retaken again from both groups.

**Data analysis**

Data from the experimental and control groups were analyzed by using SPSS, version 16. MANCOVA test was used to assess the effectiveness of the period of exercise intervention on Go-No-Go and Stroop tasks.Prior to the use of this test, the assumptions of the test were examined. Shapiro–Wilk test was used for normality of sampling distribution and Levene’s test was used for the homogeneity of variance. The analysis results showed a normal distribution of the data and equality of variances. The significance level was set at p<0.05.

## Results

**Stroop test**

**Table 2:** Stroop test measures in children with ADHD (mean± SD)

Measures		Physical activity group		Control group	
Consistent		Pre-test	Post- test	Pre-test	Post- test
	Error number	2 ± 1.94	1.05 ± 0.97	2.05±1.59	2.17 ± 1.74
	No response	2.47 ± 1.98	1.21 ± 0.91	2.76±1.39	2.58 ± 1.87
	True number	43.52 ± 3.15	45.73 ± 1.36	43.11 ±2.08	43.23±2.75
	RT(ms)	135 ± 90.26	1106.57 ± 65.77	1367.76 ± 89.65	1324.52±115.76
Inconsistent	Error number	1.52 ± 1.12	0.63 ± 1.06	1.52±1.41	1.47±1.17
	No response	4.26 ± 3.033	2.73 ± 1.04	4.52±2.71	4.52±2.26
	True number	42.10 ± 3.71	44.63 ± 1.25	41.94±3.17	42±2.73
	RT(ms)	1344.26 ± 81.51	1168.89 ± 92.76	14.17 ±75.18	1410.17±97.80
Interference		1.52 ± 1.89	1.21 ± 1.03	1.41±1.73	1.29±0.84

**Table 2** shows that the means and standard deviations of all the components of the Stroop test in the experimental group were higher than in the control group. The MANCOVA results showed that the exercise program in this research had a significant effect on the scores of the cognitive inhibition test.Wilkes λ = 0.140, F = (7, 19), P = 0.000, η² = 0.86.

The partial eta squares were equal to 0.86, which indicated that the provided exercises could explain 86% of the variance of the Stroop test scores. To determine the rate of effectiveness of exercise on Stroop test components, ANCOVA was used.

The ANCOVA test results showed that the Exercise program had the greatest impact on the RT of the consistent stimulus (P = 0.000, η² = 0.68), RT of inconsistent stimulus (P = 0.000, η² = 0.64),true number of consistent stimulus (P = 0.000, η² = 0.47), true number of inconsistent stimulus (P = 0.000, η² = 0.43),error number of consistent stimulus (P = 0.001, η² = 0.35), no response of inconsistent stimulus (P = 0.006, η² = 0.27), error number of inconsistent stimulus (P = 0.007, η² = 0.26), no response of consistent stimulus (P = 0.011, η² = 0.23), respectively. However, the interference number did not show significant changes, (P <0.05).

**Go-No-Go test**

**Table 3:** Go-No-Go test measures in children with ADHD (mean±SD)

Measures	Physical activity group		Control group	
	Pre-test	Post- test	Pre-test	Post- test
Go -True number	56.26 ± 6.72	60.47 ± 5.56	56.17 ± 7.08	56.76± 7.35
Go -Error number	13.21 ± 7.16	9 ± 5.48	13.82 ± 7.08	13.82 ± 7.08
No Go -True number	21.42 ± 2.81	24.68 ± 2.53	21± 2.76	22.11± 3.14
No Go -Error number	8.57 ± 2.81	5.31 ± 2.53	9 ± 2.76	7.88± 3.14
True RT(ms)	431.52 ± 34.22	404.57 ± 24.20	431.88 ± 35.14	420.82± 29.75
Error RT(ms)	370.10 ± 34.71	352.78 ± 30.33	365.88 ± 34.39	363.41± 30.86

All the components measured in the Go-No-Go test were better in the experimental group compared with the control group. To investigate the effect of the exercise program on the Go-No-Go test, a multivariate analysis of covariance (MANCOVA) was used. The results of this test showed that the training program presented in this study, had a significant effect on the Go-No-Go test.Wilkes λ = 0.297, F = (5, 25), P = 0.000, η² = 0.703.

The partial eta squares were equal to 0.703 and explained 70% of the variance in the behavioral inhibition test. To determine the rate of effectiveness of the exercise on Go-No-Go test components, ANCOVA was used. The ANCOVA test results showed that the Exercise program had the greatest impact on the No Go-True number (P = 0.000, η² = 0.45) and the No Go-Error number (P = 0.000, η² = 0.45),being more effective than on the True RT (P = 0.000, η² = 0.39), Error RT (P = 0.002, η² = 0.29), Go -True number (P = 0.003 ,η² = 0.27) and Go -Error number (P = 0.003, η² = 0.26), respectively (P<0.05).

## Discussion 

The purpose of this study was to measure theeffect of a selected exercise program on the cognitive and behavioral inhibition of children with ADHD,with an appropriate manipulation of the exercise intensity via control in HR and exercises appropriate to the cognitive demands. The results showed that the selected exercise program had a significant impact on the Go-No-Go and Stroop test scores,but,despite the decline, the interference number in the Stroop testdid not show significant.

Although, the mechanism between exercise and inhibition and ADHD are still not completely understood,the beneficial effects of the exercise on inhibition may be due to the electrical settings of an individual’s nerves. Helman et al. (2003) studied the effect of acute aerobic exercise on inhibition by using the event-related potential (ERP).The results showed that after 30 minutes of exercise on a treadmill, students showed a larger P3 amplitude and a shorter P3 delayed in the inconsistent stimulus compared to the consistent stimulus. Since the P3 amplitude and delay reflected the amount of allocated resources to the attention and speed of stimulus appraisal and cognitive processes, these results suggested that an exercise of a specific intensity could improve inhibition, which may be associated with changes in allocated resources and increased detection and evaluation of stimuli [**30**].

Pontifex (2013) also used the ERP and after 20 minutes of moderate-intensity aerobic exercise, children with ADHD showed similar results regarding inhibition [**18**]. The researchers suggested that one of the reasons for which exercise might be effective for EFand inhibition was that exercise positively changes the plasticity of the brain (through neurogenesis, neuroadaptation and neuroprotective processes), increases cerebral blood flow and releases catecholamines (dopamine, epinephrine, norepinephrine) that all play an essential role in the ADHD disorder. In addition, physical activity can activate areas involved in the ADHD disorder and cognitive activities, such as the prefrontal and parietal areas [**9**,**31**]. Moreover, exercise increases the level of brain derived neurotrophic factor (BDNF), which may subsequently facilitate the progression of attention, inhibition, learning and emotions [**31**]. Also BDNF helps in the release of dopamine, which is often used in the treatment of the ADHD disorder [**32**]. Therefore, exercise and physical activity can both directly and indirectly influence the catecholamines system, which is thought to have an important role in the ADHD disorder [**10**].

The results of this study were inconsistent with the results of Verret et al. (2012). They found no significant difference in the attention and inhibition of responses using a walk/not walk task after 30 sessions of physical activity on 10 children with ADHD [**13**]. This contradiction might be due to differences in the Type of Exercise training, exercise intensity or type of task used for the measurement.Verret et al. used in their studythe walk/not walk task, butthis study used thego-no-go task. The researchers suggested that one reason for the inconsistency in the findings was the lack of focus on exercise intensity, which appeared to be an important factor in the physiological responses and subsequent cognitive performance [**7**,**22**].

BDNF is a physiological response and recent theories support its role in the pathology of ADHD [**32**]. BDNF secretion is affected by exercise intensity. Reports have shown that BDNF serum was not significantly increased after a low intensity exercise [**33**], also a high-intensity exercise increased cortisol, which suppressed the production of BDNF [**34**]. 

The present findings support the effect of the designed exercises program on behavioral and cognitive inhibition in children with ADHD. According to our knowledge, there is no other published research in which the impact of physical activity on a variety of inhibitions (cognitive and behavioral) is considered in children with ADHD.

**Limitations**

There were some limitations in this study:the fact that all the participants in this study were boys and were included in three subtypes that might have affected the results. Considering the emphasis of previous studies on the assessment of the direct effect of exercise in the absence of any drug consumption during ADHD disorder [**8**,**17**], none of the participants received any medication, which was usually used in the treatment of ADHD, during or before the study.

## Conclusion 

This study showed that the selected exercise program with a controlled duration, frequency, and severity could improve the cognitive function such as cognitive and behavioral inhibition in children with ADHD.Therefore, this study had important clinical implications and, due to the beneficial effects of purposeful and organized physical activity on EF in children with ADHD, parents and schools should work together to maximize opportunities for targeted physical activities.

**Acknowledgements**

Many thanks to all the children and their families, who enthusiastically participated in this study.
